# The Impact of Macronutrient Intake on Sleep Quality in Female Endurance Athletes: A Pilot Observational Cross-Sectional Study

**DOI:** 10.3390/nu17081368

**Published:** 2025-04-17

**Authors:** Natsue Koikawa, Yume Minamino, Yu Kawasaki, Takatoshi Kasai, Yoshio Suzuki

**Affiliations:** 1 Graduate School of Health and Sports Science, Juntendo University, Chiba 270-1695, Japan; nkoikawa@juntendo.ac.jp; 2 Japanese Center for Research on Women in Sport, Juntendo University, Tokyo 113-8421, Japan; 3 Tamanoi Vinegar Co., Ltd., Osaka 590-0940, Japan; m.yume1115@gmail.com; 4 Department of Obstetrics and Gynecology, Juntendo University Graduate School of Medicine, Tokyo 113-8421, Japan; ykawasa@juntendo.ac.jp; 5 Department of Cardiovascular Biology and Medicine, Juntendo University Graduate School of Medicine, Tokyo 113-8421, Japan; 6 Sleep and Sleep-Disordered Breathing Center, Juntendo University Hospital, Tokyo 113-8421, Japan

**Keywords:** sleep quality, female endurance athletes, low energy availability, macronutrient, Pittsburgh sleep quality index, Epworth sleepiness scale, Fitbit

## Abstract

**Background/Objectives**: Sleep is essential for athletes. However, the impact of dietary habits on sleep quality in female endurance athletes at risk for low energy availability (LEA) is underexplored. This was a pilot study to examine the correlation between dietary patterns and sleep quality in healthy female endurance athletes. **Methods**: Twenty-four female endurance athletes recorded their dietary intake and sleeping habits for 6 days. Dietary intake data were collected via meal logs and photos. Sleep parameters were tracked using the Fitbit Charge 3 device. Correlation analyses were performed to explore the associations between macronutrient intake and sleep. **Results**: The athletes’ mean consumption was 2049.3 ± 396.9 kcal/day (52.9% carbohydrates, 28.2% fat, and 17.2% protein). One-third of the athletes had poor sleep quality, and thirty-eight percent experienced high daytime sleepiness. A higher protein intake was correlated with a lower awake time (R = −0.491; *p* = 0.015), and fat intake was related to a lower duration of deep sleep (R = −0.477; *p* = 0.019). Deep sleep was negatively correlated with fat intake during dinner (R = −0.417; *p* = 0.042) and was positively correlated with carbohydrate intake (R = 0.417; *p* = 0.042). **Conclusions**: In healthy female endurance athletes without LEA, dietary fat intake, especially at dinner, negatively affects deep sleep. Meanwhile, carbohydrates promote deep sleep. Therefore, optimizing macronutrient balance during evening meals may enhance sleep quality and, consequently, athletic performance.

## 1. Introduction

Female athletes encounter distinct physiological challenges compared with their male counterparts when it comes to optimizing training and performance in competitive settings. Specifically, the relationship between sleep and nutrition is complex and multifaceted. The intake of energy and macronutrients that are sources of energy affect sleep. For example, a 4-week energy restriction (800 kcal/day) decreased nighttime body temperature and slow-wave sleep and increased sleep onset latency (SOL) in nine women who were overweight [1]. Based on this finding, a lack of energy may affect sleep quality. Another study conducted on 26 healthy adults showed that saturated fat as a percentage of energy decreased the duration of slow-wave sleep in ad libitum meals of 1 day after a 4-day controlled diet [2]. In addition, a high-glycemic-index dinner eaten 4 h before bedtime decreased SOL compared with a low-glycemic-index dinner [3]. Therefore, macronutrient intake, even only at dinner, can affect sleep. However, the relationship between diet and sleep may differ between athletes and the general population. For athletes who experience continuous energy demands because of intense training, this relationship may not align with that of the general population.

Sleep is as important as training and nutrition in maintaining a good condition among athletes. In 2024, the 2023 International Olympic Committee Consensus on Relative Energy Deficiency in Sport and reviews showed that decreased sleep quality was an indicator of low energy availability (LEA) and relative energy deficiency in sport [4,5,6]. In fact, a recent study found a correlation between LEA and poor sleep quality in 42 young male rugby players [7]. However, despite the recent attention to the effects of an athlete’s diet on sleep, the number of studies that have examined the correlation between sleep quality and dietary habits in athletes is not sufficient. In particular, the correlation is important in female endurance athletes who are at high risk for LEA [8]. This is because they want to restrict their diet to control their weight to improve performance [9]. However, no studies have examined the correlation between diet and sleep quality in female endurance athletes.

Therefore, the current study aimed to evaluate the effect of dietary patterns on sleep quality in female endurance athletes.

## 2. Materials and Methods

### 2.1. Participants

Female endurance athletes who belonged to the university athletic team participated in this study. They habitually practiced for at least 2 h a day, 6 days a week. They were fully informed about the purpose and procedures of this study. The athletes or the parents or guardians of athletes aged ≤18 years provided written informed consent.

This study was approved by the Ethics Committee of the Juntendo University Faculty of Health and Sport Science (approval no.: 2021-46; approval date: 16 June 2021) and was conducted in accordance with the ethical standards of the 1964 Declaration of Helsinki and subsequent amendments, or equivalent ethical standards.

### 2.2. Experimental Design

This study was conducted during the 3-month preseason training period from August 2021 to November 2021. Each participant recorded her diet and sleep on 6 consecutive days when she was not menstruating and went to bed where she usually slept (at home). The consumption of caffeinated or alcoholic beverages was prohibited during the measurement periods.

The body composition of the participants was measured using a Bod Pod (Toyo Medic, Tokyo, Japan), which had been measured in the early morning during fasting after urinating, and the data closest to the measurement were used.

The participants filled out a survey form with information about the contents and times of their meals, including breakfast, lunch, dinner, snacks, and supplements. Simultaneously, they took photos of all the foods they ate and saved them in a designated Google Drive. Based on their records and images, the energy and macronutrient content of the diets, including snacks, beverages, and supplements, was calculated using Excel Eiyo-kun Ver. 9 (Kenpakusha, Tokyo, Japan). The energy content of the macronutrients was calculated using the Atwater conversion factor, with 4 kcal/g for carbohydrates and proteins and 9 kcal/g for fats. The mean intake for 6 days was taken as the habitual daily intake for each participant.

The participants wore the Fitbit Charge 3 device (Fitbit, San Francisco, CA, USA) to measure sleep from the time they went to bed until they woke up. The total sleep time (in min) and N3 (or deep) sleep percentage, as measured by the device, showed strong correlations with those obtained through electroencephalography, with correlation coefficients of 0.83 and 0.68, respectively [10]. However, SOL exhibited a weak correlation (correlation coefficient: r = 0.37) [10]. Thus, the SOL was not used in the current study. The SOL and the duration of awake time (minutes) after sleep onset were used as the time awake, and the time awake-to-time in bed ratio was used as the percentage awake. The total sleep time (minutes) was calculated by subtracting the time spent awake from the time spent in bed. Sleep was classified as rapid eye movement (REM) sleep (minutes), light sleep (minutes), and deep sleep (minutes). The ratio (%) of each to the total sleep time was defined as the REM, light, and deep sleep ratios. Nonetheless, the absolute values of the sleep parameters measured by the Fitbit device may not be entirely accurate. Therefore, this study focused on correlations rather than absolute values. The time from the end of dinner to the beginning of the measurement was defined as “dinner to bedtime”. The 6-day average was used as the habitual sleep time for each participant.

### 2.3. Statistical Analysis

Continuous variables were presented as the mean ± standard deviation for normally distributed data and the median and interquartile range for non-normally distributed data, and categorical variables were summarized as numbers and percentages. The normality of the continuous variables was examined using the Shapiro–Wilk test. Macronutrient intake was analyzed as the density per 1000 kcal. The normality could not be assumed for some sleep parameters and macronutrients. Therefore, the association between the variables was evaluated using the nonparametric Spearman’s correlation coefficient (R). A *p*-value of <0.05 indicated significant differences. The Statistical Package for the Social Sciences software ver. 29.0 (IBM Inc., Tokyo, Japan) was used for the analysis.

## 3. Results

### 3.1. Participants

This study enrolled all female endurance athletes in the university athletic team (N = 27). Among them, one did not complete the measurements. In addition, one participant was menstruating on the day of measurement, and another one had a history of restless leg syndrome, which was reported after joining this study. These 3 participants were excluded, and the remaining 24 participants (5 middle-distance runners, 14 long-distance runners, and 5 racewalkers) were included in the analysis. On the days of measurement, they practiced for 3.3 ± 0.6 h per day. Table 1 shows the background characteristics of the participants, including age, weight, and body fat percentage.

### 3.2. Energy and Macronutrients

The daily energy intake calculated from the records was 1983.0 (532.8) kcal. This was equivalent to 47.3 ± 11.7 kcal/kg FFM. If the daily intake was divided into breakfast, lunch, dinner, and others, the energy from breakfast was 30.7% ± 4.2%; that from lunch was 30.5% ± 4.9%; that from dinner was 35.1% ± 4.8%; and that from others was 3.7% ± 4.4%. None of the participants had anything to eat or drink after dinner.

However, because the self-reported dietary survey could have the potential for over- or underreporting, macronutrient intake was expressed in terms of energy density. Carbohydrates provided 52.9% of the total energy (Table 2).

### 3.3. Sleep Parameters

The participants went to bed 222.6 ± 41.7 min after dinner and stayed in bed for 440.7 ± 42.2 min. Of this time, they were awake for 56.0 ± 10.7 min and slept for 384.6 ± 39.1 min. If sleep was divided into subcategories, light sleep was the longest at 243 ± 31.8 min (55.3% ± 5.6%), followed by REM sleep at 73.9 ± 23.5 min (16.8% ± 5.0%) and deep sleep at 67.6 ± 15.4 min (15.3% ± 2.9%). Table 3 depicts the summary of the sleep.

### 3.4. Correlation of Nutrient Intake with Sleep Parameters

**Figure 1:**
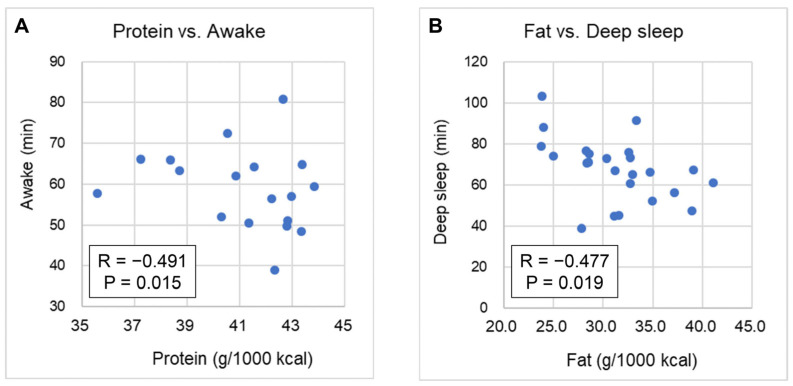
Correlation between daily macronutrient intake and sleep: (**A**) protein intake and duration of awake time; (**B**) fat intake and duration of deep sleep time.

The correlations between the macronutrient intake and sleep duration were evaluated. For daily intake, a negative correlation was observed between protein intake and time awake (R = −0.491; 95%CI: −0.752 to −0.097; *p* = 0.015) and between fat and deep sleep (R = −0.477; 95%CI: −0.744 to −0.078; *p* = 0.019; Figure 1).

**Figure 2:**
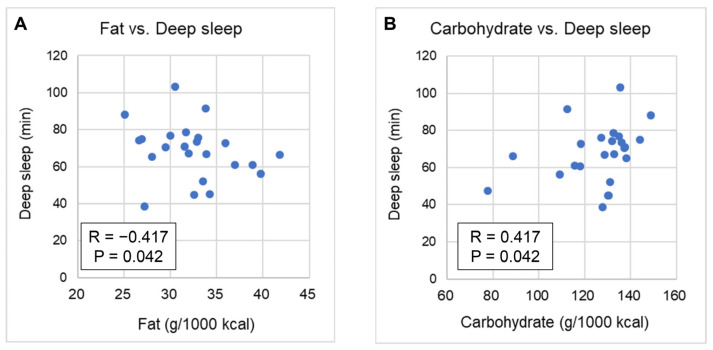
Correlation between macronutrients from dinner and sleep: (**A**) fat intake and duration of deep sleep time; (**B**) carbohydrate intake and duration of deep sleep time.

When restricted to dinner, a negative correlation was observed between deep sleep and fat intake (R = −0.417; 95% CI: −0.709 to −0.004; *p* = 0.042). Moreover, a positive correlation was observed between deep sleep and carbohydrate intake (R = 0.417; 95% CI: 0.004–0.709; *p* = 0.042; Figure 2). Supplementary Table S1 shows the overall correlation between dietary intake and sleep.

## 4. Discussion

This study investigated the influence of energy and macronutrient intake on the quality of sleep in female endurance athletes. The results show that the daily fat intake ad libitum was correlated inversely with the duration of deep sleep. Furthermore, the daily protein intake was correlated inversely with the duration of awake time. When focusing on dinner, the fat intake was correlated inversely with the duration of deep sleep. Meanwhile, the carbohydrate intake was correlated directly with the duration of deep sleep. These findings indicate that in female endurance athletes, if the daily protein intake was higher and the daily fat intake was lower, the sleep quality was better. Moreover, if the carbohydrate intake was higher and the fat intake was lower at dinner, the sleep quality was better.

At present, there is a lack of data regarding the relationship between macronutrient intake and sleep parameters in athletes. In populations not specifically comprising athletes, several studies have reported correlations between macronutrient intake, primary energy sources, and sleep. In an observational cohort of postmenopausal women, fat intake was inversely correlated with total sleep time assessed with actigraphy [11]. Using the data from the 2007–2008 NHANES, Grandner and colleagues reported that a lower carbohydrate intake was associated with difficulties in sleep maintenance [12]. In the population-based cohort, a lower carbohydrate intake was associated with greater sleep fragmentation [13]. Taken together, both carbohydrate and fat intakes are associated with sleep. However, the former is associated with good sleep and the latter with poor sleep. When focusing on nocturnal intakes, nocturnal fat intake was correlated directly with awakening after sleep onset and N2 sleep (i.e., shallow sleep) in healthy non-athletic males and females. However, it was inversely correlated with sleep efficiency and REM sleep [14]. Therefore, an increased nocturnal fat intake is correlated with poor sleep quality. The results of our study, indicating that fat intake negatively impacts sleep quality and carbohydrate intake positively influences it, are consistent with these findings. Nevertheless, the difference is that ours were assessed in female endurance athletes.

In an interventional study involving 14 young men, a high-carbohydrate intake with a low-fat diet for 2 days (600 g of carbohydrates, 75 g of proteins, and 33 g of fat) was correlated with better sleep quality, as assessed by the duration of slow-wave sleep than a balanced diet (350 g of carbohydrates, 75 g of protein, and 140 g of fat) or a low-carbohydrate intake with a high-fat diet (100 g of carbohydrates, 75 g of protein, and 255 g of fat) [15]. In a randomized controlled crossover study involving 44 men and women, Lindseth et al. found that a high-carbohydrate diet for 4 days (56% carbohydrates, 22% protein, and 20% fat) facilitated easy sleep onset. In addition, a high-protein diet (22% carbohydrates, 56% protein, and 22% fat) reduced the number of wake episodes during sleep compared with a normal diet (50% carbohydrates, 15% protein, and 35% fat) [16]. In another randomized controlled crossover study involving 36 young men and women, a reduced number of wake episodes during sleep was observed following a 4-day high-carbohydrate diet (80% carbohydrates, 10% protein, and 10% fat) [17]. Conversely, a study involving eight young healthy men showed that unlike a high-carbohydrate diet (72% carbohydrates, 16% protein, and 13% fat), a very-low-carbohydrate diet (<1% carbohydrates, 38% protein, and 61% fat) 4 h before sleep resulted in increased slow-wave sleep and decreased REM sleep [18]. Such inconsistent results regarding the effects of carbohydrate intake on sleep in interventional studies may be attributed to differences in the proportion of carbohydrates, protein, and fat in macronutrient manipulation.

Additionally, differences in the proportion of macronutrients, particularly carbohydrates and fats, between the interventional study and habitual dietary patterns may be another explanation for such inconsistency. Meanwhile, St-Onge et al. reported that in the ad libitum dinner after controlling food for 4 days, the proportion of macronutrients in the ad libitum dinner played some roles in the alterations in sleep quality [2]. This finding may be supported by our observation on the correlation between the consumption of ad libitum meals and sleep over a 6-day period. Therefore, if each individual eats a habitual fat diet ad libitum, particularly at dinner, it will have a negative effect on sleep, while nocturnal carbohydrates will have a positive effect on sleep.

Interestingly, in the interventional study conducted by Lindseth et al., a high-protein diet (56% protein) was associated with significantly fewer wake episodes than a controlled diet (15% protein) [16]. The current study showed a significant inverse correlation between daily protein intake (mean protein intake: 17.2%; range: 14.2–22.3%) and the duration of awake time. Therefore, daily protein intake can contribute to sleep maintenance even if it is not at an extreme level. As there are extremely few reports on the correlation between protein intake and sleep, further research on this topic should be performed. Nevertheless, considering the fact that our study was conducted in the female endurance athlete population, ensuring an appropriate proportion of macronutrients in the daily diet, particularly nocturnal intake (dinner) may be a promising strategy for promoting good sleep and, in turn, maintaining an overall good health condition in female endurance athletes.

Female endurance athletes are at high risk for LEA [8], and LEA, particularly LCA, is believed to affect sleep [4]. The cutoff for LEA in women is 30 kcal/kg FFM [4]. Our study was conducted based on the hypothesis that female endurance athletes are in a state of LEA, particularly LCA, and that this affects sleep. However, the energy intake of the participants in this study was 47.3 ± 11.7 kcal/kg FFM, and 52.9% (range: 44.1–61.1%) of the total energy was derived from carbohydrates. Thus, the participants in this study were not in a state of LEA or LCA, and the expected effects of LEA and LCA on sleep in female athletes could not be assessed. However, this study has shown that even in healthy female athletes without issues with their energy intake, macronutrients have an effect on sleep. Therefore, the results of this study are valuable in showing the correlation between a normal diet and sleep in healthy young women.

This pilot study had several limitations. First, it was observational in nature, and the findings were based on a cross-sectional analysis. Therefore, the results do not support a cause-and-effect relationship between macronutrients and sleep. Nevertheless, further interventional studies in female endurance athletes should be performed to validate such cause-and-effect relationships between diet and sleep. Second, sleep parameters were detected using a wearable sleep-tracking device rather than formal polysomnography. However, polysomnography may not be feasible for athletes due to time constraints, limited access, and high costs. Thus, in the current study, we specifically evaluated sleep using a simple method because an easy, inexpensive, and feasible tool is required for athletes. In addition, a wearable sleep-tracking device is better than polysomnography because it can be performed in a familiar sleep environment (i.e., at home) and can be used for assessment with a longer duration, consecutively. Furthermore, the absolute values of sleep parameters measured by a wearable device may not be entirely accurate. Therefore, this study focused on correlations rather than absolute values. Finally, the sample size of the study was relatively small. However, our sample was from a homogenous population (i.e., female endurance athletes belonging to one university athletic team). Therefore, there were minimal variations in age, body composition, daily schedule, and daily diet, and daily training levels were minimal. We believe that this is the strength of the current study.

## 5. Conclusions

In female endurance athletes, daily fat intake on an ad libitum basis may be inversely associated with the duration of deep sleep, as assessed by a sleep tracker device. Regarding nocturnal meals, fat and carbohydrate intakes may be inversely and directly associated with deep sleep duration, respectively. These findings should be confirmed through further interventional research.

## Figures and Tables

**Table 1:** Characteristics of the participants.

n = 24		Mean/Median	SD/IQR	Min.	Max.
Age	Years	19.5	0.9	18	21
Type of endurance sports					
Long-distance	n, %	14 (58.3)			
Middle-distance	n, %	5 (20.8)			
Racewalk	n, %	5 (20.8)			
Hours of practice per day	h	3.3	0.6	2.5	4.5
Height	cm	161.9	4.9	152.3	170.0
Body weight	kg	51.1	5.4	40.9	61.3
BMI	kg/m^2^	19.4	1.2	17.1	22.8
Fat-free mass	kg	43.8	4.0	38.6	50.4
Fat	%	14.2 ^†^	25.7 ^†^	5.6	17.8

BMI = body mass index; ^†^ median (IQR).

**Table 2:** Energy and macronutrient intake of the participants.

		Dairy Intake				Breakfast (%)		Lunch (%)		Dinner (%)		Others (%)	
Nutrients		Mean/Median	SD/IQR	Min.	Max.	Mean	SD	Mean	SD	Mean	SD	Mean	SD
Energy	kcal	1983.0 ^†^	532.8 ^†^	1545.3	3396.1	30.7	4.2	30.5	4.9	35.1	4.8	3.7	4.4
Protein	g ^1^	43.1	4.3	35.6	55.7	31.9	5.3	29.5	5.3	35.9	5.8	2.7	3.4
	%	17.2	1.7	14.2	22.3								
Fat	g ^1^	31.4	4.8	23.8	41.1	30.7	5.3	29.4	6.4	37.0	5.2	2.9	5.6
	%	28.2	4.3	21.4	36.9								
Carbohydrate	g ^1^	132.2	11.7	110.2	152.7	30.7	5.3	31.1	5.8	33.8	6.9	4.5	6.0
	%	52.9	4.7	44.1	61.1								

^1^ g per 1000 kcal; ^†^ median (IQR).

**Table 3:** Summary of the participants’ sleeping habits.

Conditions		Mean/Median	SD/IQR	Min.	Max.
Dinner to bedtime	min/day	222.6	41.7	127.0	321.0
Duration in bed	min/day	440.7	42.2	363.8	520.2
Awake	min/day	56.0	10.7	39.0	80.8
	% ^1^	14.9 ^†^	16.4 ^†^	9.8	40.7
Total sleep time	min/day	384.6	39.1	306.0	462.2
REM sleep	min/day	73.9	23.5	35.7	123.3
	% ^2^	16.8	5.0	8.1	26.2
Light sleep	min/day	243.1	31.8	188.8	307.5
	% ^2^	55.3	5.6	43.3	67.6
Deep sleep	min/day	67.6	15.4	38.7	103.2
	% ^2^	15.3	2.9	9.8	20.8

^1^ % represents awake (min)/duration in bed (min), ^2^ % represents the subcategory of sleep (min)/sleep (min), and ^†^ median (IQR).

## Data Availability

The datasets analyzed during the current study are not publicly available because of personal information. The data supporting the findings of this study are available from the corresponding author upon reasonable request.

## Supplementary Materials

The following supporting information can be downloaded at https://www.mdpi.com/article/10.3390/nu17081368/s1: Table S1. Correlations between sleep parameters and macronutrient intake.

## Abbreviations

The following abbreviations were used in this manuscript:

IOC	International Olympic Committee
LEA	Low energy availability
REM	Rapid eye movement
SOL	Sleep onset latency
