# Supplementary material for: The Impact of Macronutrient Intake on Sleep Quality in Female Endurance Athletes: A Pilot Observational Cross-Sectional Study
Source: Nutrients. 2025 Apr 17;17(8):1368. doi: 10.3390/nu17081368 (PMC12030440; doi:10.3390/nu17081368)
Supplement: Supplementary file 1 [file nutrients-17-01368-s001.zip › nutrients-3558369-supplementary.pdf]

Table S1. Correlations between sleep parameter and macronutrient intake

a. Speamans correlation coefficiet between sleep parameter and nutrient intake

	Daily intake			Intake from dinner		
	Protein	Fat	Carbohydrate	Protein	Fat	Carbohydrate
Sleep (min)	0.248	-0.098	0.043	0.065	-0.160	0.180
Awake (min)	-0.491	-0.247	0.381	-0.122	-0.257	0.245
Awake (%)	-0.337	0.085	0.033	0.020	-0.059	0.048
REM sleep (min)	0.350	0.060	-0.128	0.145	-0.072	0.143
Light sleep (min)	0.077	-0.069	0.044	0.010	-0.035	0.034
Deep sleep (min)	0.359	-0.477	0.344	0.095	-0.417	0.417
REM sleep ratio (%)	0.323	0.137	-0.205	0.187	-0.011	0.043
Light sleep ratio (%)	-0.230	0.063	0.018	-0.121	0.150	-0.159
Deep sleep ratio (%)	0.270	-0.369	0.260	0.144	-0.318	0.297

Cells were gradiently colored according to the values; blue (-1) to red (1).

b. P values of the correlation

	Daily intake			Intake from dinner		
	Protein	Fat	Carbohydrate	Protein	Fat	Carbohydrate
Sleep (min)	0.243	0.648	0.840	0.762	0.455	0.400
Awake (min)	0.015	0.245	0.066	0.571	0.226	0.248
Awake (%)	0.108	0.692	0.880	0.926	0.785	0.824
REM sleep (min)	0.094	0.779	0.550	0.500	0.739	0.506
Light sleep (min)	0.719	0.750	0.837	0.965	0.872	0.875
Deep sleep (min)	0.085	0.019	0.099	0.660	0.042	0.042
REM sleep ratio (%)	0.123	0.525	0.336	0.382	0.958	0.843
Light sleep ratio (%)	0.281	0.771	0.933	0.574	0.485	0.458
Deep sleep ratio (%)	0.203	0.076	0.220	0.503	0.130	0.158

P < 0.05 is highlighted.
